# Knowledge of nursing staff before and after training on incontinence-associated dermatitis*

**DOI:** 10.1590/1980-220X-REEUSP-2023-0272en

**Published:** 2024-03-29

**Authors:** Raelly Ramos Campos Ximenes, Edna Maria Camelo Chaves, Ana Lívia Araújo Girão, Maria Helane Rocha Batista Gonçalves, Saionara Leal Ferreira, Rhanna Emanuela Fontenele Lima de Carvalho

**Affiliations:** 1 Universidade Estadual do Ceará, Programa de Pós-Graduação em Cuidados Clínicos em Enfermagem e Saúde, Fortaleza, CE, Brazil.; 2 Instituto Dr. José Frota, Fortaleza, CE, Brazil.; 3 Hospital Geral Dr. Waldemar Alcântara, Fortaleza, CE, Brazil.; 4 Universidade Federal do Ceará, Hospital Universitário Walter Cantídio, Fortaleza, CE, Brazil.

**Keywords:** Pressure Ulcer, Dermatitis, Fecal Incontinence, Urinary Incontinence, Enterostomal Therapy, Úlcera por Presión, Dermatitis, Incontinencia Fecal, Incontinencia Urinaria, Estomaterapia

## Abstract

**Objective:**

To verify the knowledge of nursing staff before and after training on incontinence-associated dermatitis.

**Method:**

A study before and after an educational intervention carried out with nursing staff from the medical and surgical clinics and intensive care unit of the university hospital in June 2023. The training took place over three meetings. Data was collected using a questionnaire administered immediately before and after the training. McNemar’s test for dependent samples was used to compare before and after training.

**Results:**

25 nurses and 14 nursing technicians took part. The items that showed statistical significance were related to the identification and correct differentiation of dermatitis associated with incontinence and pressure injury; and the correct way to sanitize the skin.

**Conclusion:**

The training of the nursing team made it possible to assess their knowledge of how to identify, prevent and treat incontinence-associated dermatitis.

## RESUMEN

**Objetivo:**

Verificar los conocimientos del personal de enfermería antes y después de la formación sobre la dermatitis asociada a la incontinencia.

**Método:**

Estudio antes y después de una intervención formativa realizada con personal de enfermería de las clínicas médicas y quirúrgicas y de la unidad de cuidados intensivos de un hospital universitario en junio de 2023. La formación se impartió en tres sesiones. Los datos se recogieron mediante un cuestionario aplicado inmediatamente antes y después de la formación. Se utilizó la prueba de McNemar para muestras dependientes para comparar antes y después de la formación.

**Resultados:**

Participaron 25 enfermeras y 14 técnicos de enfermería. Los ítems que mostraron significación estadística estaban relacionados con la identificación y correcta diferenciación de la dermatitis asociada a la incontinencia y al daño por presión; y la forma correcta de higienizar la piel.

**Conclusión:**

La formación del equipo de enfermería permitió evaluar los conocimientos del equipo de enfermería sobre cómo identificar, prevenir y tratar la dermatitis asociada a la incontinencia.

## INTRODUCTION

Incontinence-associated dermatitis (IAD) is a common ailment in patients with fecal and/or urinary incontinence. It may cause discomfort, pain, burning, itching or tingling in the affected areas. Clinical signs include erythema (ranging from pink to red), a whitish appearance and swelling of the surrounding skin (indicating maceration) and poorly demarcated borders^(1)^.

Pressure Injury (PI), on the other hand, is damage to the skin due to intense and/or prolonged pressure on a bony prominence or related to the use of medical devices, often confused with IAD in the early stages^(2)^. However, IAD is a risk factor for PI, which is considered a preventable adverse event^(3,4)^.

IAD has a significant impact on the health of hospitalized patients. In a survey of 5,342 adult patients in intensive care units in 36 states in the United States (USA), it was found that more than a third of patients – 46.6% – had incontinence of urine, feces or both. The overall prevalence of IAD was 21.3%; the prevalence of IAD among patients with incontinence was 45.7%. Just over half of the IADs were classified as mild (52.3%), moderate (27.9%) and severe (9.2%). The prevalence of PI in the sacral region among individuals with incontinence was 17.1%. Multivariate analysis revealed that both the presence of IAD and immobility were associated with a significantly increased likelihood of developing PI in the sacrum^(5)^. The total hospital cost index becomes 1.2 times higher for incontinent patients and 1.3 times higher for patients being treated with IAD^(3)^.

The prevention and treatment strategies for IAD and PI are different. The nursing team plays a key role in the prevention and proper management of IAD, through the implementation of specific measures such as the use of appropriate barrier products, hygiene, humidity and incontinence control, as well as education and guidance for patients and caregivers^(6)^.

By understanding the differences between IAD and PI, nurses can identify and intervene early, adopting specific approaches for each condition. In addition, nursing staff should be aware of the risk factors associated with both conditions, such as immobility, poor nutrition, advanced age and diaper use, in order to implement appropriate preventive strategies and ensure quality care for patients^(2)^.

Therefore, early detection, prevention and treatment of IAD requires nursing professionals to understand the physiological aspects of the skin in order to correctly identify and differentiate skin lesions, as well as intensifying care through the nursing process, linked to evidence-based protocols^(7)^.

In this sense, it is necessary to develop permanent education actions to improve care practices by professionals in relation to IAD^(8)^. This includes the implementation of courses and in-service training. The implementation of educational programs brings a positive attitude, as it presents an adequate level of knowledge for pillars in care, as well as basic patient safety precepts directed at the subject^(9)^.

It is essential that nurses improve their knowledge and add it to the team, in search of care based on scientific evidence. Health professionals must seek qualifications, but health institutions must provide training to generate new ideas and exchange experiences, with quality time for this^(10)^.

The nursing team’s lack of knowledge on the subject of the assessment, prevention and classification of PI is linked to the quality of care provided. Therefore, this shows that professionals need qualifications to improve their knowledge. It should also be noted that nursing is an indispensable part of a multi-professional team, which continues to provide care to health users and is committed to providing qualified care with scientific and technical knowledge^(10)^.

Although there are studies on the identification, prevention and treatment of IAD, there is a lack of national scientific publications on this subject. In this way, investigating the knowledge of the nursing team, considering the national reality, will provide an overview of how this population knows about IAD.

In this way, the importance and relevance of this work to empower scientific research and improve the knowledge of nursing professionals about IAD and its aspects for detection, proper management and prevention can be seen. “Such statements are of great importance to subsidize and assist in the design of comprehensive and effective health care^(11)^.”

In order to contribute to the consolidation of knowledge regarding care for IAD, the aim of this study was to verify the knowledge of the nursing team before and after training on incontinence-associated dermatitis.

The research hypothesis tested was: the proportion of right and wrong answers is different before and after training.

## METHOD

### Type of Study

Quasi-experimental, before-and-after study.

### Site

The research was conducted at a University Hospital in the city of Fortaleza – Ceará, administered by the Brazilian Hospital Services Company (EBSERH), a public company governed by private law, linked to the Ministry of Education, with the aim of providing medical and hospital care services.

### Study Sample

The type of sample was by convenience, with the following inclusion criteria: being a nursing professional providing direct care to patients in the medical and surgical units and the Intensive Care Unit (ICU) linked to the study hospital. The exclusion criterion was not answering one of the pre-test or post-test questionnaires.

A total of 42 professionals took part in the course. Of these, 3 professionals were excluded (2 nurses and 1 nursing technician) for not completing the pre- and/or post-tests. Thus, the final study sample consisted of 39 professionals who took part in the research: 25 nurses (64.1%) and 14 nursing technicians (35.9%).

### Data Collection and Study Period

The topic of the training was Safe Care in the Prevention and Treatment of Incontinence-Associated Dermatitis. The content covered was: Identification of IAD, Risk factors, Prevention and Treatment of IAD, Difference between PI and IAD, Notification and Indicators of IAD.

This course was planned in April 2023 and came live into the virtual environment platform of the Electronic Information System (SEI), with a project describing the training plan and a letter of consent from the institution’s nursing inpatient unit coordinator for release.

Once the course had been assessed, approved and cleared for implementation by the institution’s People Development Unit, a registration link was created for participants via the EBSERH Corporate Education School Platform (3EC), which aims to promote training for network employees.

It was publicized by the nursing units’ immediate supervisors in the sectors’ WhatsApp groups. Thus, all the nursing staff, nurses and nursing technicians in the sectors were invited to take part in the training, but were informed that participation in the survey would be optional.

Three classes were offered, two online and one face-to-face. The face-to-face class took place in a room at the institution, in the afternoon from 2pm to 3.45pm. The two synchronous online classes (using Microsoft Teams) took place in the evening, from 7.15pm to 9pm, on different dates. Each class had 25 places available for nursing professionals, nurses and nursing technicians, and nursing residents, with a timetable of 1 hour 45 minutes. The course was taught by three nurses, the coordinator and two facilitators, and took place in June 2023.

The Informed Consent Form (ICF), pre-test and post-test instruments were registered on the 3EC Platform and google forms to assess the nursing team’s knowledge of IAD.

The data collection instrument (pre- and post-test) on the team’s knowledge related to IAD was previously evaluated in its content by two specialist nurses, one in stomatherapy and the other in dermatology, with extensive practical experience in the area.

The questionnaire consisted of two parts: the first refers to the participants’ characterization data (age, gender, work unit, professional category, qualifications and length of training); and the second part contains 14 items. The first two items deal with identifying and recognizing the difference between DAI and LP. The answers to these first two items were yes or no. The rest of the items ask questions about the identification, prevention and treatment of IAD, with right or wrong answers^(11a)^.

Before starting the training, the professionals who wanted to take part in the research were asked to sign the ICF. The researcher then sent the link to the pre-test and, after the training, the link to the post-test. The link to access the instruments was made available on the 3EC Platform and on Google Forms via Microsoft Teams chat. Only after signing the informed consent form did the researcher obtain the data relating to the study.

At the end of the training, the Reaction Assessment was applied via the link sent by UDP in the online class, and in the face-to-face class it was via QR Code. It is worth noting that all participants received a digital certificate of participation from the institution via the 3EC Platform, regardless of whether or not they had taken part in the research by completing the pre- and post-test instrument.

### Data Analysis and Processing

The research results were exported to the Statistical Package for the Social Sciences (SPSS) software, version 20.0. McNemar’s test for dependent samples was used to compare the groups before and after the training. A p-value of <0.05 was considered.

### Ethical Aspects

The research was cleared in 2022 by the Research Ethics Committee of the State University of Ceará (UECE) under opinion no. 5.268.049 and the Walter Cantídio University Hospital/Federal University under opinion no. 5.288.935, in accordance with Resolution no. 466 of December 12, 2012.

## RESULTS

Table 1 of the total number of participants, 35 (89.74%) were female, with an average age of 40. As for length of training, 31 (79.49%) had been working for more than 10 years. In terms of qualifications, 22 (56.41%) had a specialization degree. As for their work unit, 19 (48.72%) were from surgical wards.

**Table 1:** Sociodemographic characteristics of the nursing team participating in the training – Fortaleza, CE, Brazil, 2023.

Variable	n (%)
**Professional category**	
Nurse	25 (64,1)
Nursing technician	14 (35,9)
**Gender**	
Female	35 (89,74)
Male	4 (10,26)
**Degree**	
Specialization	22 (56,41)
Technical specialization	13 (33,33)
Master’s degree	4 (10,26)
**Length of training**	
>15 years	16 (41,03)
Between 10 – 15 years	15 (38,46)
Between 6 – 10 years	6 (15,38)
< 5 years	2 (5,13)
**Work unit**	
Surgical ward	19 (48,72)
Medical ward	15 (38,46)
Intensive Care Unit	5 (12,82)

There was a statistically significant difference (p < 0.05) between the pre- and post-test answers to the first two questions: 100% of the professionals said in the post-test that they knew how to identify and differentiate IAD from PI (X2 = 6.125; p = 0.008). Two other items showed a statistically significant difference when compared before and after the training: items 8 and 9. Item 8: “When cleaning the skin, liquid soap with an acid pH should be preferred” (X2 = 13.067; p = 0.001), and item 9: “In the absence of suitable products for cleaning skin exposed to humidity, it is preferable to clean the skin with soap and water” (X2 = 17.053; p = 0.001). (Table 2)

**Table 2:** Number of right and wrong answers in the pre-test and post-test and comparison using McNemar’s test – Fortaleza, CE, Brazil, 2023.

Questions about incontinence-associated dermatitis	Pre-test n(%)		Post-test n(%)		
	yes	no	yes	no	p-value
1. Do you know how to identify incontinence-associated dermatitis?	31 (79,5)	8 (20,5)	39 (100)	–	**0,008**
2. Do you know the difference between pressure injuries and incontinence-associated dermatitis?	32 (82,1)	7 (17,9)	39 (100)	–	
	**Rights**	**Wrongs**	**Rights**	**Wrongs**	**p-value**
3. Incontinence-Associated Dermatitis (IAD) is a clinical manifestation characterized by erythema and edema of the skin surface, which may be accompanied by flictenas, erosion or secondary skin infection, common in patients with urinary and/or fecal incontinence. (R)	36 (92,3)	3 (7,7)	38 (97,4)	1 (2,6)	0,625
4. IAD results from pressure on a bony prominence or associated with the location of a medical device. (W)	37 (94,9)	2 (5,1)	38 (97,4)	1 (2,6)	1
5. It is not possible to confuse IAD with pressure injuries in the early stages. (W)	33 (84, 6)	6 (15,4)	33 (84, 6)	6 (15,4)	1
6. Non-blanchable erythema or non-reactive hyperemia is characteristic of IAD. (R)	27 (69,2)	12 (30,8)	28 (71,8)	11 (28,2)	1
7. The most common sites of IAD are: groin, intergluteal region, buttocks, labia majora in women, and testicles in men. (R)	39 (100)	–	39 (100)	–	
8. When sanitizing the skin, liquid soap with an acidic pH should preferably be used. (R)	8 (20,5)	31 (79,5)	23 (59,0)	16 (41,0)	**0,001**
9. In the absence of suitable products for cleaning skin exposed to humidity, it is preferable to clean the skin with soap and water. (W)	4 (10,3)	35 (89,7)	23 (59,0)	16 (41,0)	**0,001**
10. IAD without the presence of infection is classified as 1A and 2A according to GLOBIAD. (R)	30 (76,9)	9 (23,1)	36 (92,3)	3 (7,7)	0,07
11. In IAD category 1A, skin protection (barrier cream or spray) should be applied frequently according to the manufacturer’s instructions to patients with urinary and/or fecal incontinence. (R)	36 (92,3)	3 (7,7)	38 (97,4)	1 (2,6)	0,625
12. Request an opinion from a dermatology or stomatherapy specialist nurse who is part of the work institution’s skin committee for an individualized assessment whenever there is loss of epidermis – category 2 of the RTI. (R)	35 (89,7)	4 (10,3)	38 (97,4)	1 (2,6)	0,25
13. Changes in the color of the skin around the lesion are not factors that help to differentiate between PI and IAD. (W)	29 (74,4)	10 (25,6)	32 (82,1)	7 (17,9)	0,581
14. Skin protectors can come in the form of creams, pastes, films or lotions. It is recommended to apply the skin protectant frequently according to the manufacturer’s instructions. (R)	37 (94,9)	2 (5,1)	38 (97,4)	1 (2,6)	1

IAD: Incontinence-Associated Dermatitis; PI: Pressure injuries; GLOBIAD: The Ghent Global IAD Categorisation Tool.

Table 2 shows the number of hits and misses for each item in the pre-test and post-test.

## DISCUSSION

The results of this study concur with those of a study^(12)^ on IAD carried out with 30 nursing professionals from a university hospital, the majority of whom were female nurses aged between 30 and 39.

The aim of this study was to train nurses and nursing technicians through continuing education, so that they are able to identify and prevent IAD. In the legal sphere, the Federal Nursing Council (COFEN), a federal authority that regulates nursing practice, regulates the care of patients with wounds, through Resolution No. 567, of January 29, 2018, stating that nurses, technicians and nursing assistants must keep up to date by participating in continuing education programs^(13)^.

A Brazilian study^(9)^ evaluated the knowledge of nursing professionals in a medical clinic unit about the skin conditions IAD and PI, with the participation of nurses (59%) and nursing technicians (57%), who mentioned that training in health services improves the knowledge of nursing teams regarding the early identification of changes related to IAD and PI.

On the topics of identification and the difference between IAD and PI, the vast majority of participants got it right in the pre- and post-test. In contrast to the findings of a study^(14)^ which aimed to assess nurses’ knowledge of IAD in order to understand the extent of this problem in a teaching hospital, the results found regarding knowledge of IAD identification showed that nurses know the definition, but were wrong to attribute the clinical identification of PI to IAD, demonstrating difficulty in differentiating between the two types of lesions.

Fragile knowledge on the part of the multi-professional team, especially nursing professionals, is a risk factor for the development and inappropriate management of cases of IAD, since they have difficulty differentiating between other types of lesions, such as PI^(9)^.

Item 8 was statistically significant, p = 0.001, with 8 (20.5%) people answering true in the pre-test, but in the post-test, the correct answer was 23 (59.0%). Item 9 showed statistical significance with p = 0.001, in which 4 (10.3%) people answered the item as false (but it was the correct answer), but in the post-test, the hit rate was 23 (59.0%) for this item.

It can be seen that one question complemented the other. The literature states that the pH of healthy skin is between 5.0 and 5.5, so it is beneficial to choose a cleaning agent with a low pH, to use mild and non-irritating surfactants, soft cloths, and it is prudent to avoid alkaline products, such as soaps, which can change the pH of the skin surface to a more basic environment, promoting bacterial growth^(15,16,17)^.

“In addition, cleansing using mechanical movements and alkaline pH soaps can lead to skin breakdown by removing its natural lipids, which serve as a protective barrier. Cleaning should preferably be carried out with liquid soaps with a neutral or acidic pH, but as most conventional soaps have an alkaline pH, the use of rinse-free cleaning agents with an acidified pH has been recommended^(8)^.”

It can be seen that after the explanations of the ideal product for cleaning the skin, there was an improvement in the number of correct answers. However, these products are not always available in health services, so it was emphasized that on this occasion it is preferable to use only water.

Regarding the treatment approach in category 1A, item 11, there were a significant number of correct answers, which indicates the team’s knowledge of IAD treatment in this category. A study^(18)^ underlines the advice that for patients with Category 1 IAD (red and intact skin), in addition to gentle cleansing, it is recommended to use an acrylate terpolymer film or a product based on petrolatum or containing dimethicone.

With regard to the type of composition of the products, item 14, used both for the prevention and treatment of IAD, it is of the utmost importance to be familiar with the various types of skin protectors and their form of presentation available in the institution, as this facilitates nursing care for patients affected by IAD.

Research^(19)^ points out that when carefully assessing the skin and identifying patients most at risk of IAD, skin cleansing should be carried out, and barrier products containing petrolatum and dimethicone, zinc oxide-based creams, liquid acrylate film should be used, as they have a moisturizing and barrier function, although there is still no consensus on the best product to use.

Concerning the treatment of IAD in category 2, item 12, the products used in the treatment were covered in the training. However, as the institution has a team of nurses who specialize in skin care, it was advised that if there are any doubts about the treatment, the experts should be asked for their opinion in order to assess and guide the topical conduct, since the humidity of IAD can be a risk factor for developing PI, and so proper monitoring and management would prevent this adverse event.

This guidance is in line with a study^(7)^ which showed that 86% of nurses have the knowledge to manage mild IAD and moderate or severe cases and to manage this dermatological complication process and differentiate it from stage 1 PI. However, in category 2 IAD, the nurse should ask the institution’s skin lesions/healing commission to evaluate and guide appropriate conduct in handling this category.

Still on the treatment of category 2 IAD, if signs of infection are observed, it is indicated to take a sample for microbiological analysis and the result should be used to decide on the therapy (e.g. antifungal cream, antibiotic, anti-inflammatory product)^(18)^.

However, the training reinforced that the decision on medication is a medical matter, and the nurse can point out what they have identified in the skin assessment and discuss the best course of action, putting it in writing on the medical prescription (by the doctor) so that the nursing team can follow it appropriately.

At the end of the training, there was a reaction survey. Participants considered that the content was applicable to their professional reality and that they had gained new knowledge. The findings were “corroborated by a recent study^(12)^, which also ran a course on IAD through the social network Instagram and, after the final evaluation and the feedback received by the course participants, it was noticeable that the action managed to get good adherence from the participants, with several positive comments, demonstrating that educational actions can be conducted through unconventional means, such as social media, so commonly used by people for communication and leisure time.”

In another study on the same subject^(20)^, it was observed that the educational intervention provoked reflections based on practice, but based on scientific knowledge, and led to changes in the group involved, with a better understanding of the subject and assertive decision-making.

In item 13, when highlighting factors to differentiate PI and IAD, even though there was no statistical significance, 10 (25.6%) of the participants marked it as right (wrong item). The differential diagnosis between PI and IAD is based on visual examination and patient history^(21)^. Incorrect classification has significant implications for prevention, treatment and comparative assessment of quality of care, as suggested by other studies^(22)^. In addition, IAD has been reported as PI, negatively impacting institutional epidemiological indicators.

The investigation of the nursing team’s knowledge was adopted because of the need to identify whether health professionals are able to identify and prevent IAD in care practice, and training in the workplace as continuing education, updating the team on appropriate and current care, thus enriching clinical nursing care for this issue.

It should be noted that this research had limitations in terms of the number of participants and the fact that the research was carried out at a single educational institution. It should be noted that the 3EC platform is used for courses offered at the institution and is a recently introduced technology. This limited the number of participants, as some professionals found it difficult to register for the course and download the Microsoft Teams application to attend online classes.

## CONCLUSION

Regarding the tested hypothesis, the proportion of right and wrong answers is different before and after the training, with only items 8 and 9 obtaining statistical significance, it can be seen that there was a significant improvement in the correct answers in the post-test. It is known that not everyone is aware of the pathophysiology of IAD and, consequently, the clinical reasoning for using suitable products for skin hygiene. However, this training provided an environment for exchanging knowledge and updating content not only on IAD, but also on PI, which has an impact on care practice.

Although the other items were not statistically significant, there was an improvement in the number of correct answers in the post-test, showing that doubts or difficulties presented before the training began had repercussions on the increase in the number of correct answers in the post-test. Therefore, the proportion of true and false answers was different before and after the training.

Given the need for the nursing team to be able to correctly identify, prevent and treat IAD, continuing education in service is important.
