# ERRATUM: Knowledge of nursing staff before and after training on incontinence-associated dermatitis

**DOI:** 10.1590/1980-220X-REEUSP-2023-0272eren

**Published:** 2024-06-17

In the article “Knowledge of nursing staff before and after training on incontinence-associated dermatitis”, with the DOI: https://doi.org/10.1590/1980-220X-REEUSP-2023-0272en published by the journal: “Revista da Escola de Enfermagem da USP, volume 58 of 2024:e20230272, p.1-7, on page 3:

**Where was written:**

The questionnaire consisted of two parts: the first refers to the participants’ characterization data (age, gender, work unit, professional category, qualifications and length of training); and the second part contains 14 items. The first two items deal with identifying and recognizing the difference between DAI and LP. The answers to these first two items were yes or no. The rest of the items ask questions about the identification, prevention and treatment of IAD, with right or wrong answers.

**Now read:**

The questionnaire consisted of two parts: the first refers to the participants’ characterization data (age, gender, work unit, professional category, qualifications and length of training); and the second part contains 14 items. The first two items deal with identifying and recognizing the difference between DAI and LP. The answers to these first two items were yes or no. The rest of the items ask questions about the identification, prevention and treatment of IAD, with right or wrong answers^(11a)^.

On page 6:

**Now read:**

After reference 11, add:

11a. Sokem JAS, Ferreira AM, Bergamaschi FPR, Coelho MMF, Rigotti, MA, Carneiro LM. Construction and validation of a tool for evaluation of knowledge about incontinence-associate dematitis. Enferm Glob. 2022;21(68):56-70. doi: https://doi.org/10.6018/eglobal.519901.

