# Supplementary material for: Knowledge of nursing staff before and after training on incontinence-associated dermatitis
Source: Rev Esc Enferm USP. 2024 Mar 29;58:e20230272. doi: 10.1590/1980-220X-REEUSP-2023-0272en (PMC11021019; doi:10.1590/1980-220X-REEUSP-2023-0272en)
Supplement: Translation

# Translation: Conhecimento da equipe de enfermagem antes e após capacitação sobre dermatite associada à incontinência*

**DOI:** 10.1590/1980-220X-REEUSP-2023-0272pt

**Authors:** Raelly Ramos Campos Ximenes, Edna Maria Camelo Chaves, Ana Lívia Araújo Girão, Maria Helane Rocha Batista Gonçalves, Saionara Leal Ferreira, Rhanna Emanuela Fontenele Lima de Carvalho

**Affiliations:** 1 Universidade Estadual do Ceará, Programa de Pós-Graduação em Cuidados Clínicos em Enfermagem e Saúde, Fortaleza, CE, Brasil.; 2 Instituto Dr. José Frota, Fortaleza, CE, Brasil.; 3 Hospital Geral Dr. Waldemar Alcântara, Fortaleza, CE, Brasil.; 4 Universidade Federal do Ceará, Hospital Universitário Walter Cantídio, Fortaleza, CE, Brasil.

**Keywords:** Úlcera por Pressão, Dermatite, Incontinência Fecal, Incontinência Urinária, Estomaterapia

## Abstract

**Objetivo:**

Verificar o conhecimento da equipe de enfermagem antes e após capacitação sobre dermatite associada à incontinência.

**Método:**

Estudo antes e depois de uma intervenção educativa realizado com profissionais da equipe de enfermagem das clínicas médicas, cirúrgicas e unidade de terapia intensiva do hospital universitário, no mês de junho de 2023. A capacitação ocorreu durante três encontros. Os dados foram coletados por meio de questionário, aplicado imediatamente antes e após a capacitação. Para a comparação do antes e após capacitação, foi realizado o teste de *McNemar* para amostras dependentes.

**Resultados:**

Participaram 25 enfermeiros e 14 técnicos de enfermagem. Os itens que apresentaram significância estatística foram relacionados à identificação e à diferenciação correta da dermatite associada à incontinência e lesão por pressão; e a forma correta para higienização da pele.

**Conclusão:**

A capacitação da equipe de enfermagem permitiu avaliar o conhecimento da equipe de enfermagem para identificar, prevenir e tratar a dermatite associada à incontinência.

## INTRODUÇÃO

A Dermatite Associada à Incontinência (DAI) é uma condição comum em pacientes com incontinência fecal e/ou urinária. Pode causar desconforto, dor, queimação, prurido ou formigamento nas áreas afetadas. Os sinais clínicos incluem eritema (variando de rosa para vermelho), aparência esbranquiçada e inchaço da pele circundante (indicando maceração) e bordas mal demarcadas^(1)^.

A Lesão por Pressão (LP), por sua vez, é um dano na pele devido à pressão intensa e/ou prolongada em uma proeminência óssea ou relacionada ao uso de dispositivos médicos, muitas vezes confundida com DAI nos estágios iniciais^(2)^. No entanto, a DAI é fator de risco para LP, que é considerada um evento adverso passível de prevenção^(3,4)^.

A DAI possui um impacto significativo na saúde dos pacientes internados. Em pesquisa em 36 estados dos Estados Unidos (EUA) com 5.342 pacientes adultos em unidades de cuidados intensivos, viu-se que mais de um terço dos pacientes 46,6% apresentavam incontinência de urina, fezes ou ambos. A prevalência geral de DAI foi de 21,3%; a prevalência de DAI entre pacientes com incontinência foi de 45,7%. Pouco mais da metade das DAI foi classificada como leve, com 52,3%; moderada, com 27,9%; e considerada grave, com 9,2%. A prevalência de LP na região sacral entre indivíduos com incontinência foi de 17,1%. A análise multivariada revelou que tanto a presença de DAI quanto imobilidade foram associados a uma probabilidade significativamente aumentada de desenvolver LP sacral^(5)^. O índice total de custos hospitalares se torna 1,2 vezes maior para pacientes incontinentes e 1,3 vezes maior para pacientes em tratamento com DAI^(3)^.

As estratégias de prevenção e tratamento para DAI e LP são diferentes. A equipe de enfermagem desempenha um papel fundamental na prevenção e no gerenciamento adequado da DAI, por meio da implementação de medidas específicas, como a utilização de produtos de barreira adequados, higiene, controle da umidade e da incontinência, além de educação e orientação aos pacientes e cuidadores^(6)^.

Ao compreender as diferenças entre a DAI e a LP, os enfermeiros podem identificar e intervir precocemente, adotando abordagens específicas para cada condição. Além disso, a equipe de enfermagem deve estar ciente dos fatores de risco associados a ambas as condições, como imobilidade, má nutrição, idade avançada e uso de fraldas, a fim de implementar estratégias preventivas adequadas e garantir um cuidado de qualidade aos pacientes^(2)^.

Sendo assim, detecção, prevenção e tratamento precoces da DAI requer dos profissionais de enfermagem compreensão sobre os aspectos fisiológicos da pele para correta identificação e diferenciação de lesões de pele, além de intensificação dos cuidados por meio do processo de enfermagem, atrelados a protocolos baseados em evidências^(7)^.

Nesse sentido, é necessário desenvolver ações de educação permanente para aperfeiçoar práticas de cuidado pelos profissionais em relação a DAI^(8)^. Isso inclui a implementação de cursos e treinamentos em serviço. A implementação de programas educativos, traz uma atitude positiva, pois apresenta um nível de conhecimento adequado para as bases do atendimento, além de preceitos básico de segurança do paciente direcionado ao tema^(9)^.

É fundamental que o enfermeiro aprimore seus conhecimentos e os agregue à equipe, em busca de um cuidado baseado em evidências científicas. Os profissionais de saúde devem buscar qualificações, no entanto, as instituições de saúde devem disponibilizar treinamentos que gerem novas ideias, troca de experiências, com tempo de qualidade para isso^(10)^.

O déficit de conhecimento da equipe de enfermagem acerca do tema em relação à avaliação, à prevenção e à classificação das LP está ligado à qualidade da assistência prestada. Portanto, isso mostra que profissionais têm necessidade de qualificações para aprimorarem seus conhecimentos. Destaca-se, ainda, que a enfermagem é parte indispensável de uma equipe multiprofissional, que continua atuando na assistência ao usuário de saúde e tem o compromisso de prestar uma assistência qualificada e com conhecimentos científicos e técnicos^(10)^.

Apesar de existirem estudos sobre identificação, prevenção e tratamento da DAI, existe carência de publicação científica nacional relativa a essa temática. Dessa forma, investigar o conhecimento da equipe de enfermagem, considerando a realidade nacional, fornecerá um panorama de como está o conhecimento desta população acerca da DAI.

Desta forma, percebe-se a importância e relevância deste trabalho para empoderar as pesquisas científicas e aprimorar os conhecimentos dos profissionais de enfermagem sobre a DAI e seus aspectos para detecção, gerenciamento adequado e prevenção. “Tais colocações são de grande importância para subsidiar e auxiliar no traçar de uma assistência à saúde de forma ampla e eficaz^(11)^.”

No sentido de contribuir para consolidação do conhecimento referente aos cuidados assistenciais para a DAI, o objetivo desse estudo foi verificar o conhecimento da equipe de enfermagem antes e após capacitação sobre dermatite associada à incontinência.

A hipótese de pesquisa testada foi: a proporção de respostas verdadeira e falsa é diferente antes e após a capacitação.

## MÉTODO

### Tipo de Estudo

Estudo quase-experimental, do tipo antes e depois.

### Local

A pesquisa foi realizada em um Hospital Universitário na cidade de Fortaleza – Ceará, administrado pela Empresa Brasileira de Serviços Hospitalares (EBSERH), empresa pública de direito privado, vinculada ao Ministério da Educação, com a finalidade de prestar serviços de assistência médico-hospitalar.

### Amostra do Estudo

A amostra foi por conveniência, com os seguintes critérios de inclusão: ser profissional de enfermagem de assistência direta a pacientes nas unidades de clínicas médicas, cirúrgicas e Unidade de Terapia Intensiva (UTI) vinculados ao hospital do estudo. Como critério de exclusão, não responder a um dos questionário de pré-teste ou pós-teste.

Participaram do curso, 42 profissionais. Destes, 3 profissionais foram excluídos (2 enfermeiras e 1 técnica de enfermagem) por não preencherem o pré e/ou pós testes. Assim, a amostra final do estudo foi de 39 profissionais que participaram da pesquisa: 25 enfermeiros (64,1%) e 14 técnicos de Enfermagem (35,9%).

### Coleta de Dados e Período do Estudo

A capacitação teve como tema Cuidado Seguro na Prevenção e Tratamento em Dermatite Associada à Incontinência. O conteúdo abordado foi: Identificação da DAI, Fatores de risco, Prevenção e Tratamento da DAI, Diferença de LP × DAI, Notificação e Indicadores da DAI.

Este curso foi planejado no mês de abril de 2023 e inserido na plataforma de ambiente virtual do Sistema Eletrônico de Informações (SEI), com um projeto com descrição do planejamento da capacitação e ofício de anuência da coordenadora de unidade de internação da enfermagem da instituição para liberação.

Após avaliação, aprovação e liberação para execução do curso pela Unidade de Desenvolvimento de Pessoas da instituição, foi criado um *link* de inscrição para os participantes pela Plataforma da Escola EBSERH de Educação Corporativa (3EC), que visa promover a capacitação dos colaboradores da rede.

A divulgação foi realizada por meio das chefias imediatas de enfermagem das unidades nos grupos de WhatsApp dos setores. Assim, todos os profissionais da equipe de enfermagem, enfermeiros e técnicos de enfermagem dos setores, foram convidados a participar da capacitação, porém foi informado que seria opcional a participação na pesquisa.

Foram ofertadas três turmas, duas em modalidades *on-line* e uma presencial. A turma presencial ocorreu em uma sala na instituição, turno da tarde de 14 horas às 15h45min. As duas turmas ofertadas na modalidade *on-line* síncrono (pelo *Microsoft Teams*), ocorreram no turno da noite, das 19h15min às 21 horas, em datas distintas. Cada turma teve disponibilidade para 25 vagas para profissionais de enfermagem, enfermeiros e técnicos de enfermagem, e residentes de enfermagem, tendo carga horária de 1h45min. O curso foi ministrado por três enfermeiras, a coordenadora e duas facilitadoras, e ocorreu no mês de junho de 2023.

Foram cadastrados os instrumentos de Termo de Consentimento Livre Esclarecido (TCLE), pré-teste e pós-teste na Plataforma 3EC e no *google forms*, para avaliação do conhecimento da equipe de enfermagem para DAI.

O instrumento de coleta de dados (pré e pós-teste) sobre o conhecimento da equipe relacionado a DAI, foi avaliado previamente em seu conteúdo por duas enfermeiras especialistas, uma em estomaterapia e outra em dermatologia, com vasta experiência prática na área.

O questionário é composto por duas partes: a primeira refere-se aos dados de caracterização dos participantes (idade, sexo, unidade de trabalho, categoria profissional, titulação e tempo de formação); e segunda parte contém 14 itens. Os dois primeiros itens versam sobre a identificação e reconhecimento da diferença entre DAI e LP. As respostas para esses dois primeiros itens era sim ou não. O restante dos itens aborda perguntas sobre identificação, prevenção e tratamento da DAI com respostas verdadeira ou falsa^(11a)^.

Antes de iniciar a capacitação, para os profissionais que quisessem participar da pesquisa, foi solicitado que assinassem o TCLE. Logo em seguida, a pesquisadora enviou o *link* do pré-teste, e após a capacitação, o *link* do pós-teste. *O link* para acesso dos instrumentos foi disponibilizado pela Plataforma 3EC e no *Google Forms* através do *chat* no *Microsoft Teams*. Somente após a assinatura do TCLE, a pesquisadora obteve os dados relativos ao estudo.

Ao final da capacitação foi aplicada a Avaliação da Reação através do *link* enviado pela UDP na aula *on-line,* e na aula presencial foi através de QR Code. Vale ressaltar que todos os participantes receberam o certificado digital de participação pela instituição através da Plataforma 3EC, independentemente de ter participado ou não da pesquisa preenchendo o instrumento pré e pós-teste.

### Análise e Tratamento dos Dados

Os resultados da pesquisa foram exportados para o Programa *Software Statistical Package for the Social Sciences* (SPSS), versão 20.0. Para a comparação dos grupos antes e após a capacitação foi realizado o teste de *McNemar* para amostras dependentes. Considerou-se o valor de p < 0,05.

### Aspectos Éticos

A pesquisa foi aprovada em 2022 pelo Comitê de Ética em Pesquisa da Universidade Estadual do Ceará (UECE) sob parecer nº 5.268.049 e do Hospital Universitário Walter Cantídio/ Universidade Federal sob parecer nº 5.288.935, em conformidade com a Resolução nº 466 de 12 de dezembro de 2012.

## RESULTADOS

Na Tabela 1, dos participantes, 35 (89,74%) eram do sexo feminino, com idade média de 40 anos. Quanto ao tempo de formação, 31 (79,49%) atuavam há mais de 10 anos. No tocante a titulação, 22 (56,41%) possuíam especialização. Em relação a unidade de trabalho, 19 (48,72%) eram das clínicas cirúrgicas.

**Tabela 1:** Características sociodemográficas da equipe de enfermagem participante da capacitação – Fortaleza, CE, Brasil, 2023.

Variável	n (%)
**Categoria profissional**	
Enfermeiro(a)	25 (64,1)
Técnico(a) de enfermagem	14 (35,9)
**Sexo**	
Feminino	35 (89,74)
Masculino	4 (10,26)
**Titulação**	
Especialização	22 (56,41)
Especialização de nível técnico	13 (33,33)
Mestrado	4 (10,26)
**Tempo de formação**	
>15 anos	16 (41,03)
Entre 10 – 15 anos	15 (38,46)
Entre 6 – 10 anos	6 (15,38)
< 5 anos	2 (5,13)
**Unidade de trabalho**	
Clínica cirúrgica	19 (48,72)
Clínica médica	15 (38,46)
Unidade de Terapia Intensiva	5 (12,82)

Observou-se que houve diferença estatisticamente significante (p < 0,05) entre as respostas no pré e pós-teste nas duas primeiras perguntas: 100% dos profissionais afirmaram no pós-teste que saberiam identificar e diferenciar a DAI da LP (X^2^= 6,125; p = 0,008). Dois outros itens apresentaram diferença estatisticamente significante quando comparado antes e após a capacitação: itens 8 e 9. Item 8: “Na higienização da pele deve-se usar preferencialmente sabonete líquido com pH ácido” (X^2^= 13,067; p = 0,001), e o item 9: “Na falta de produtos adequados para realizar a limpeza de pele exposta a umidade, é preferível higienizar a pele com água e sabão” (X^2^= 17,053; p = 0,001). (Tabela 2)

**Tabela 2:** Número de acertos e erros no pré-teste e pós-teste e comparação com uso do teste de *McNemar* – Fortaleza, CE, Brasil, 2023.

Perguntas sobre dermatite associada à incontinência	Pré-teste n(%)		Pós-teste n(%)		
	sim	não	sim	não	p
1. Você sabe identificar a dermatite associada à incontinência?	31 (79,5)	8 (20,5)	39 (100)	–	**0,008**
2. Você sabe a diferença de lesão por pressão e dermatite associada à incontinência?	32 (82,1)	7 (17,9)	39 (100)	–	
	**Acertos**	**Erros**	**Acertos**	**Erros**	**p**
3. A Dermatite Associada à Incontinência (DAI) é uma manifestação clínica caracterizada por eritema e edema da superfície da pele, pode ser acompanhada por flictenas, erosão ou infecção cutânea secundária, comum em pacientes com incontinência urinária e/ou fecal. (V)	36 (92,3)	3 (7,7)	38 (97,4)	1 (2,6)	0,625
4. A DAI resulta de uma pressão sobre a proeminência óssea ou associada à localização de um dispositivo médico. (F)	37 (94,9)	2 (5,1)	38 (97,4)	1 (2,6)	1
5. Não é possível confundir DAI com lesão por pressão em estágios iniciais. (F)	33 (84, 6)	6 (15,4)	33 (84, 6)	6 (15,4)	1
6. O eritema não branqueável ou hiperemia não reativa é característica da DAI. (F)	27 (69,2)	12 (30,8)	28 (71,8)	11 (28,2)	1
7. Os locais mais comuns de aparecer a DAI são: virilhas, região interglútea, nádegas, grandes lábios em mulheres, e testículos em homem. (V)	39 (100)	–	39 (100)	–	
8. Na higienização da pele deve-se usar, preferencialmente, sabonete líquido com pH ácido. (V)	8 (20,5)	31 (79,5)	23 (59,0)	16 (41,0)	**0,001**
9. Na falta de produtos adequados para realizar a limpeza de pele exposta a umidade, é preferível higienizar a pele com água e sabão. (F)	4 (10,3)	35 (89,7)	23 (59,0)	16 (41,0)	**0,001**
10. A DAI sem presença de infecção é classificada como 1A e 2A de acordo com a GLOBIAD. (V)	30 (76,9)	9 (23,1)	36 (92,3)	3 (7,7)	0,07
11. Na categoria 1A da DAI, deve-se aplicar o protetor de pele (creme de barreira ou spray), com frequência de acordo com indicação do fabricante, em pacientes com incontinência urinária e/ou fecal. (V)	36 (92,3)	3 (7,7)	38 (97,4)	1 (2,6)	0,625
12. Solicitar parecer de enfermeira especialista em dermatologia ou estomaterapia que faça parte da comissão de pele da instituição de trabalho para avaliação individualizada sempre que houver perda de epiderme – categoria 2 da DAI. (V)	35 (89,7)	4 (10,3)	38 (97,4)	1 (2,6)	0,25
13. As alterações na coloração da pele ao redor da lesão não são fatores que ajudam a diferenciar LP e DAI. (F)	29 (74,4)	10 (25,6)	32 (82,1)	7 (17,9)	0,581
14. Protetores de pele podem vir na forma de cremes, pastas, películas ou loções. Recomenda-se aplicar o protetor de pele com frequência de acordo com as instruções do fabricante. (V)	37 (94,9)	2 (5,1)	38 (97,4)	1 (2,6)	1

DAI: Dermatite Associada à Incontinência; LP: Lesão por pressão; GLOBIAD: The Ghent Global IAD Categorisation Tool.

Na Tabela 2, está descrito o número de acertos e erros para cada item do pré-teste e pós-teste.

## DISCUSSÃO

Os resultados do presente estudo corrobora com uma pesquisa^(12)^, com DAI, realizada com 30 profissionais de enfermagem de um hospital universitário, em que a maioria eram enfermeiros, do sexo feminino e idade entre 30 e 39 anos.

Nesta pesquisa buscou-se capacitar enfermeiros e técnicos de enfermagem, por meio da educação permanente, para que estejam aptos a identificar e prevenir a DAI. No âmbito legal, o Conselho Federal de Enfermagem (COFEN), autarquia federal que regulamenta a atuação da enfermagem, normatiza o cuidado aos pacientes com ferida, por meio da Resolução Nº 567, de 29 de janeiro de 2018, afirmando que enfermeiros, técnicos e auxiliares de enfermagem devem manter-se atualizados participando de programas de educação permanente^(13)^.

Um estudo^(9)^ brasileiro avaliou o conhecimento dos profissionais de enfermagem da unidade de clínica médica sobre as afecções cutâneas DAI e LP, contando com a participação de enfermeiros (59%) e técnicos de Enfermagem (57%), os quais mencionaram que a capacitação em serviços de saúde, melhora o conhecimento das equipes de enfermagem quanto à identificação precoce das alterações relacionadas a DAI e LP.

Nos tópicos de identificação e diferença entre a DAI e LP, os participantes em sua grande maioria acertaram no pré e pós-teste. A confrontação com os achados^(14)^ que teve como objetivo avaliar o conhecimento de enfermeiros sobre DAI com o intuito de entender a amplitude desta problemática em um hospital de ensino, os resultados encontrados acerca do conhecimento sobre identificação de DAI demonstraram que os enfermeiros conhecem a definição, contudo, erraram ao atribuir a identificação clínica de LP à DAI, demonstrando dificuldade de diferenciar os dois tipos de lesões.

A fragilidade de conhecimento por parte da equipe multiprofissional, sobretudo dos profissionais de enfermagem, é fator de risco para o desenvolvimento e conduta inadequada dos casos de DAI, uma vez que demonstram dificuldade acerca da diferenciação em relação a outros tipos de lesões, como por exemplo, a LP^(9)^.

O item 8 apresentou significância estatística, p = 0,001, com 8 (20,5%) pessoas que responderam verdadeiro no pré-teste, mas no pós-teste, o índice de acerto foi para 23 (59,0%). O item 9 apresentou significância estatística com p = 0,001, em que 4 (10,3%) pessoas responderam o item como falso (porém era a resposta correta), mas no pós-teste, o índice de acerto foi para 23 (59,0%) para este item.

Percebe-se que uma questão complementava a outra. Na capacitação, foi enfatizado sobre o pH levemente ácido da nossa pele, a literatura afirma que o pH da pele saudável fica entre 5,0 e 5,5 portanto, é benéfico a escolha de um agente de limpeza com pH baixo, utilizar surfactantes suaves e pouco irritantes, panos macios, sendo prudente evitar produtos alcalinos, como sabonetes/sabão, que podem alterar o pH da superfície da pele para um ambiente mais básico, promovendo o crescimento bacteriano^(15–17)^.

“Ademais, a higienização por meio de movimentos mecânicos, juntamente com sabonetes de pH alcalino, pode levar à ruptura da pele pela remoção de seus lipídios naturais, que servem como barreira protetora. A limpeza deve ser realizada, preferencialmente, com sabonetes líquidos de pH neutro ou ácido, porém, como a maioria dos sabonetes convencionais apresentam pH alcalino, tem sido recomendado o uso de agentes de limpeza sem enxágue e com pH acidificado^(8)^.”

Destarte percebe-se que após as explicações do produto ideal para higienização da pele houve melhora nos acertos. No entanto, nem sempre estes produtos são disponibilizados nos serviços de saúde, então, foi enfatizado que nesta ocasião é preferível utilizar apenas água.

Na abordagem do tratamento na categoria 1A, item 11, foi verificado importante número de acertos, o que denota conhecimento da equipe no tratamento da DAI nesta categoria. Estudo^(18)^ reforça a orientação que para pacientes com DAI Categoria 1 (pele vermelha e íntegra), é recomendado além da limpeza suave usar filme de terpolímero de acrilato ou produto à base de petrolato ou contendo dimeticona.

No que se refere ao tipo de composição dos produtos utilizados tanto para prevenção quanto para o tratamento da DAI (item 14), é de suma importância que se conheça os variados tipos de protetores cutâneos e a sua forma de apresentação disponível na instituição, pois facilita a conduta de enfermagem nos cuidados assistenciais ao paciente acometido por DAI.

Pesquisa^(19)^ aponta que na avaliação cuidadosa da pele e na identificação dos pacientes em maior risco para DAI, deve-se realizar limpeza de pele, e utilizar produtos de barreira contendo petrolato e dimeticona, cremes à base de óxido de zinco, filme líquido de acrilatos, pois tem função hidratante e de barreira, não havendo, ainda, consenso sobre o melhor produto a ser aplicado.

No tocante ao tratamento da DAI na categoria 2 (item 12), foi abordado na capacitação os produtos utilizados no tratamento, no entanto, pelo fato da instituição ter equipe de enfermeiros especialistas no cuidado com a pele, foi orientado que no caso de dúvidas quanto ao tratamento seja solicitado o parecer às especialistas para avaliação e direcionamento da conduta tópica, visto que a umidade da DAI pode ser um fator de risco para desenvolver LP e, assim, o acompanhamento e condução adequada evitaria este evento adverso.

Esta orientação vai ao encontro de uma pesquisa^(7)^ que evidenciou que 86% dos enfermeiros possuem conhecimento para manejar a DAI leve e os casos moderados ou severos e gerenciar este processo de complicação dermatológica e diferenciar de LP estágio 1. Entretanto, em DAI categoria 2, o enfermeiro assistencial deve solicitar a comissão de lesões de pele/curativos da instituição avaliação e orientação de condutas adequadas no manuseio desta categoria.

Ainda sobre o tratamento da DAI categoria 2, se observados sinais de infecção, é indicada a coleta de uma amostra para análise microbiológica e o resultado deve ser usado para decidir sobre a terapia (por exemplo, creme antifúngico, antibiótico, produto anti-inflamatório)^(18)^.

Contudo, na capacitação foi reforçado que a decisão de medicamentos é conduta médica, e o enfermeiro pode sinalizar o que identificou na avaliação de pele e discutir sobre a melhor conduta, sendo colocado por escrito na prescrição médica (pelo médico) para que a equipe de enfermagem siga de forma adequada.

Ao final da capacitação, houve pesquisa de reação. Foi considerado pelos participantes aplicabilidade do conteúdo à realidade profissional e obtenção de novos conhecimentos. Os achados foi “corroborado por estudo recente^(12)^, que também realizou um curso sobre DAI, pela rede social *Instagram*, e, após a avaliação final e o *feedback* recebido pelos cursistas, foi perceptível que a ação conseguiu obter boa adesão dos participantes, com diversos comentários positivos, demonstrando que ações educativas podem ser conduzidas por meios não convencionais, como uma mídia social, tão utilizada comumente pelas pessoas para comunicação e momentos de lazer.”

Em outro estudo na mesma temática^(20)^, observou-se que a intervenção educativa provocou reflexões, a partir da prática, porém, pautadas no conhecimento científico, e suscitou mudanças no grupo envolvido, com uma melhor compreensão do tema e tomada de decisão assertivas.

No item 13, ao ressaltar fatores para diferenciar LP e DAI, mesmo não apresentado significância estatística, 10 (25,6%) dos participantes assinalaram como verdadeiro (item incorreto). O diagnóstico diferencial entre LP e DAI é baseado no exame visual e histórico do paciente^(21)^. A classificação incorreta tem implicações significativas na prevenção, tratamento e avaliação comparativa da qualidade de cuidado, assim como sugerem outros estudos^(22)^. Além de que a DAI tem sido notificada como LP, impactando negativamente nos indicadores epidemiológicos institucionais.

A investigação do conhecimento da equipe de enfermagem foi adotada pela necessidade de identificar se os profissionais de saúde estão aptos para identificar e prevenir a DAI na prática assistencial, e a capacitação em ambiente de trabalho como educação permanente, atualizando a equipe nos cuidados adequados e atuais, enriquecendo, dessa forma, os cuidados clínicos de enfermagem para esta temática.

Elucida-se que esta pesquisa teve limitações quanto a quantidade de participantes e a realização da pesquisa em uma única instituição de ensino. Destaca-se que o uso da plataforma 3EC é utilizada para cursos ofertados na instituição e é uma tecnologia recentemente instituída. Dessa forma, limitou o número de participantes, pois alguns profissionais tiveram dificuldade de acesso para inscrição do curso e baixar o aplicativo *Microsoft Teams* para assistir aulas *on-line*.

## CONCLUSÃO

No tocante a hipótese testada, a proporção de respostas verdadeira e falsa é diferente antes e após a capacitação. Mesmo que apenas os itens 8 e 9 obtiveram significância estatística, percebe-se que houve melhora significativa nos acertos no pós-teste. Sabe-se que não é de conhecimento de todos a fisiopatologia da DAI e consequentemente o raciocínio clínico para uso de produtos adequados para higienização da pele. Contudo, esta capacitação permitiu um ambiente de troca de conhecimentos e atualizações de conteúdo não somente da DAI, mas também da LP, que impacta na prática assistencial.

Embora os demais itens não apresentaram significância estatística, percebe-se melhora de acertos das respostas no pós-teste, demonstrando que dúvidas ou dificuldades apresentadas antes do início da capacitação, repercutiram no aumento do quantitativo de acertos no pós-teste. Portanto, a proporção de respostas verdadeira e falsa foi diferente antes e após a capacitação.

Perante a necessidade de que a equipe de enfermagem esteja apta para a correta identificação, prevenção e tratamento em DAI, é possível concluir na importância da educação permanente em serviço.
