# Supplementary material for: ERRATUM: Knowledge of nursing staff before and after training on incontinence-associated dermatitis
Source: Rev Esc Enferm USP. 2024 Jun 17;58:e20230272er. doi: 10.1590/1980-220X-REEUSP-2023-0272eren (PMC11245280; doi:10.1590/1980-220X-REEUSP-2023-0272eren)
Supplement: Translation

# Translation: ERRATA: Conhecimento da equipe de enfermagem antes e após capacitação sobre dermatite associada à incontinência

No artigo “Conhecimento da equipe de enfermagem antes e após capacitação sobre dermatite associada à incontinência”, com número de DOI: https://doi.org/10.1590/1980-220X-REEUSP-2023-0272pt, publicado na Revista da Escola de Enfermagem da USP, volume 58 de 2024:e20230272, p.1-7, na página 1:

**Onde se lê:**

*Extraído dada tese “Desenvolvimento de protocolo de prevenção e de tratamento para dermatite associada à incontinência”, Universidade Estadual do Ceará, 2023.

**Leia-se:**

*Extraído da tese “Desenvolvimento de protocolo de prevenção e de tratamento para dermatite associada à incontinência”, Universidade Estadual do Ceará, 2023.

Na página 3:

**Onde se lê:**

O questionário é composto por duas partes: a primeira refere-se aos dados de caracterização dos participantes (idade, sexo, unidade de trabalho, categoria profissional, titulação e tempo de formação); e segunda parte contém 14 itens. Os dois primeiros itens versam sobre a identificação e reconhecimento da diferença entre DAI e LP. As respostas para esses dois primeiros itens era sim ou não. O restante dos itens aborda perguntas sobre identificação, prevenção e tratamento da DAI com respostas verdadeira ou falsa.

**Leia-se:**

O questionário é composto por duas partes: a primeira refere-se aos dados de caracterização dos participantes (idade, sexo, unidade de trabalho, categoria profissional, titulação e tempo de formação); e segunda parte contém 14 itens. Os dois primeiros itens versam sobre a identificação e reconhecimento da diferença entre DAI e LP. As respostas para esses dois primeiros itens era sim ou não. O restante dos itens aborda perguntas sobre identificação, prevenção e tratamento da DAI com respostas verdadeira ou falsa^(11a)^.

Na página 7:

**Leia-se:**

Após a referência 11, acrescentar:

11a. Sokem JAS, Ferreira AM, Bergamaschi FPR, Coelho MMF, Rigotti, MA, Carneiro LM. Construction and validation of a tool for evaluation of knowledge about incontinence-associate dematitis. Enferm Glob. 2022;21(68):56–70. doi: https://doi.org/10.6018/eglobal.519901.
